# The Genetic Architecture of Arsenic Metabolism Efficiency:A SNP-Based Heritability Study of Bangladeshi Adults

**DOI:** 10.1289/ehp.1408909

**Published:** 2015-03-13

**Authors:** Jianjun Gao, Lin Tong, Maria Argos, Molly Scannell Bryan, Alauddin Ahmed, Muhammad Rakibuz-Zaman, Muhammad G. Kibriya, Farzana Jasmine, Vesna Slavkovich, Joseph H. Graziano, Habibul Ahsan, Brandon L. Pierce

**Affiliations:** 1 Department of Public Health Sciences, and; 2 Department of Human Genetics, The University of Chicago, Chicago, Illinois, USA; 3 UChicago Research Bangladesh (URB), Dhaka, Bangladesh; 4 Department of Environmental Health Sciences, Mailman School of Public Health, Columbia University, New York, New York, USA; 5 Comprehensive Cancer Center, and; 6 Department of Medicine, The University of Chicago, Chicago, Illinois, USA

## Abstract

**Background:**

Consumption of arsenic-contaminated drinking water adversely affects health. There is interindividual variation in arsenic metabolism efficiency, partially due to genetic variation in the arsenic methyltransferase (*AS3MT*) gene region.

**Objectives:**

The goal of this study was to assess the overall contribution of genetic factors to variation in arsenic metabolism efficiency, as measured by the relative concentration of dimethylarsinic acid (DMA%) in urine.

**Methods:**

Using data on genome-wide single nucleotide polymorphisms (SNPs) and urinary DMA% for 2,053 arsenic-exposed Bangladeshi individuals, we employed various SNP-based approaches for heritability estimation and polygenic modeling.

**Results:**

Using data on all participants, the percent variance explained (PVE) for DMA% by all measured and imputed SNPs was 16% (*p* = 0.08), which was reduced to 5% (*p* = 0.34) after adjusting for *AS3MT* SNPs. Using information on close relatives only, the PVE was 63% (*p* = 0.0002), but decreased to 41% (*p* = 0.01) after adjusting for *AS3MT* SNPs. Regional heritability analysis confirmed 10q24.32 (*AS3MT*) as a major arsenic metabolism locus (PVE = 7%, *p* = 4.4 × 10^–10^), but revealed no additional regions. We observed a moderate association between a polygenic score reflecting elevated DMA% (composed of thousands of non-*AS3MT* SNPs) and reduced skin lesion risk in an independent sample (*p* < 0.05). We observed no associations for SNPs reported in prior candidate gene studies of arsenic metabolism.

**Conclusions:**

Our results suggest that there are common variants outside of the *AS3MT* region that influence arsenic metabolism in Bangladeshi individuals, but the effects of these variants are very weak compared with variants near *AS3MT*. The high heritability estimates observed using family-based heritability approaches suggest substantial effects for rare variants and/or unmeasured environmental factors.

**Citation:**

Gao J, Tong L, Argos M, Scannell Bryan M, Ahmed A, Rakibuz-Zaman M, Kibriya MG, Jasmine F, Slavkovich V, Graziano JH, Ahsan H, Pierce BL. 2015. The genetic architecture of arsenic metabolism efficiency: a SNP-based heritability study of Bangladeshi adults. Environ Health Perspect 123:985–992; http://dx.doi.org/10.1289/ehp.1408909

## Introduction

Arsenic contamination of drinking water is a major public health problem in many countries, with > 137 million people in > 70 countries estimated to be exposed (International Agency for Research on Cancer 2004). Chronic exposure to arsenic has been linked to a wide array of health conditions (Rahman et al. 2009), including cancers of the lung, bladder, liver, kidney, and skin (Celik et al. 2008; Liu and Waalkes 2008; Mink et al. 2008; Yu et al. 2006; Yuan et al. 2010). Arsenic has also been associated with diabetes and cardiovascular disease, as well as neurological, reproductive, and respiratory conditions (Abhyankar et al. 2012; Golub et al. 1998; Huang et al. 2011; National Research Council 1999; Parvez et al. 2010; Vahidnia et al. 2007). Skin lesions are one of the earliest and most prevalent clinical manifestations of arsenic exposure and are considered the classical sign of arsenic toxicity (Yoshida et al. 2004).

Arsenic consumed in drinking water enters the blood stream as inorganic arsenic (iAs) [i.e., arsenite (As^III^) and arsenate (As^V^)] and is metabolized primarily in the liver. According to the classical Challenger model of arsenic metabolism (Rehman and Naranmandura 2012), As^III^, the predominant form of iAs in Bangladesh, is methylated using arsenic (+3 oxidation state) methyltransferase (AS3MT) as the key enzyme and *S*-adenosylmethionine (SAM) as the methyl donor (Thomas et al. 2007) to produce monomethylarsonic acid (MMA^V^). After the reduction of MMA^V^ to monomethylarsonous acid (MMA^III^), a second methylation step produces dimethylarsinic acid (DMA^V^). Some DMA^V^ can then be reduced to DMA^III^ (Thomas et al. 2004, 2007). The sum of urinary arsenic species (iAs, MMA, and DMA, including As^III^ and As^V^, MMA^III^, and MMA^V^ as well as DMA^III^ and DMA^V^) is regarded as a biomarker of recent inorganic arsenic exposure (Biggs et al. 1997), and the composition of urinary arsenic metabolites relative to total arsenic is believed to reflect arsenic methylation capacity. Higher arsenic methylation capacity is associated with lower risk for arsenical skin lesions, the classical sign of arsenic toxicity (Ahsan et al. 2007; Gao et al. 2011; Kile et al. 2011; Lindberg et al. 2007; Pierce et al. 2013; Valenzuela et al. 2005).

Familial aggregation and heritability analyses of arsenic metabolic profiles suggest that genetic factors influence interindividual variation in arsenic methylation capacity (Chung et al. 2002; Tellez-Plaza et al. 2013). Candidate gene association studies have implicated single nucleotide polymorphisms (SNPs) in the *AS3MT* gene region in arsenic methylation capacity (Agusa et al. 2011; Rodrigues et al. 2012; Schläwicke Engström et al. 2009), and a recent genome-wide association study (GWAS) confirmed this finding, showing two clear association signals in the *AS3MT* region (Pierce et al. 2012, 2013). In the GWAS, *AS3MT* was the only region in the genome that harbored variants showing associations of genome-wide significance. It remains unclear whether other SNPs that did not surpass the genome-wide significance threshold have weaker associations with arsenic methylation capacity.

In this study, we searched for evidence that additional genetic variants (other than the known *AS3MT* variants) influence arsenic methylation capacity, measured as the relative concentration of DMA in urine, using various approaches to evaluate polygenic susceptibility. We used SNP-based heritability methods to estimate the heritability in arsenic metabolism efficiency that is attributable to measured and imputed genome-wide SNPs, which we also refer to as the percent variance explained (PVE) by measured SNPs. We also used a “family-based” version of this method to estimate the full narrow-sense heritability, which reflects the additive contributions of all variants, including unmeasured rare variants (Yang et al. 2010; Zhou et al. 2013). We also conducted regional heritability analyses to estimate the heritability due to common SNPs in each segment of the genome (Nagamine et al. 2012). We used polygenic scoring (Purcell et al. 2007) to assess the polygenic contribution of arsenic metabolism variants that passed a significance threshold to skin lesion risk. In addition, we evaluated associations of 20 SNPs reported to be associated with arsenic methylation capacity in prior candidate gene studies.

## Materials and Methods

*Study population*. The Health Effects of Arsenic Longitudinal Study (HEALS) is a large prospective cohort study of the health consequences of arsenic exposure. Details of the study design have been published previously (Ahsan et al. 2006a). A total of 11,746 healthy married adults (18–75 years of age) were enrolled in 2000–2002. At baseline, study interviewers collected information on demographic and lifestyle characteristics, conducted clinical examinations, and obtained biospecimens (blood and urine). Water samples from all 5,966 wells serving the 25-km^2^ study area were collected. Follow-up surveys and comprehensive physical examinations are conducted every 2 years. Approximately 1,000 of the HEALS subjects in this analysis were randomly selected to have their metabolites measured, and > 1,000 additional participants had metabolite data available due to prior ancillary studies. Only HEALS samples were used for the primary analyses described below, including chip heritability, regional heritability, and associations for candidate SNPs. For the polygenetic scoring analyses, in addition to all 2,053 HEALS samples with metabolite data, which constituted the training set, HEALS also contributed 1,285 controls and 24 skin lesion cases to the “testing” set.

The Bangladesh Vitamin E and Selenium Trial (BEST) is a 2 × 2 factorial randomized chemoprevention trial evaluating the effects of vitamin E and selenium supplementation on nonmelanoma skin cancer risk (Argos et al. 2013). A total of 7,000 individuals have been randomized to one of four treatment arms: vitamin E only (100 IU/day), *L*-selenomethionine only (200 μg/day), both vitamin E and selenium, and placebo. All participants were required to have existing arsenic-related skin lesions to be eligible. BEST participants are residents of roughly the same geographic area as HEALS, and the studies have very similar protocols, questionnaires, and biospecimen collection procedures. Biological samples, including blood and urine, were collected at baseline, along with clinical and covariate data. In this study, 1,990 BEST participants living in the Araihazar area were randomly selected for genotyping. These 1,990 skin lesions cases were included in the polygenic scoring analyses only, as a part of the “testing set.”

*SNP genotyping*. A sample of 5,499 individuals was selected from HEALS (*n* = 3,454) and BEST (*n* = 2,045) for genome-wide SNP genotyping using Illumina’s Cyto12 SNP array (~ 300,000 SNPs). For HEALS, DNA was extracted from clotted blood using Flexigene DNA kits (catalog no. 51204; from QIAGEN. For BEST, DNA was extracted from whole blood using the QIAamp 96 DNA Blood Kit (catalog no. 51161; QIAGEN). Genotyping methods and quality control have been described previously (Pierce et al. 2012, 2013). Genotyping was conducted in two batches. A total of 5,354 participants and 257,747 SNPs passed our quality control (QC) filters. QC included sample-level filters (excluding samples with call rate < 0.97, outlying heterozygosity values, and sex mismatches) and marker-level filters (excluding SNPs with call rates < 0.95 and Hardy–Weinberg *p* < 10^–10^, and minor allele frequency < 0.01) as described previously (Pierce et al. 2012, 2013). The total genotyping rate among eligible samples was 99.8%. Genotype imputation was conducted using MaCH (Markov Chain Haplotyping algorithm) software and the HapMap 3 GIH reference panel (Gujarati Indians in Houston), yielding genotypes for 1,211,988 SNPs after QC, restricting to SNPs with an imputation accuracy of *r*^2^ > 0.3 (Li et al. 2010).

*Measurements of arsenic in water and urine*. Urinary arsenic was measured at the Trace Metals Core Laboratory at Columbia University, which is a member of the QC program run by the Institute de Sante Publique du Quebec and uses their QC samples to standardize the measurements of urinary arsenic. The laboratory has consistently measured urinary arsenic concentration with correlation > 0.97 for blinded quality control samples. Urinary creatinine was measured by a colorimetric diagnostics kit (Sigma). The sum of urinary arsenic concentration was divided by creatinine to obtain creatinine-adjusted total arsenic concentration (micrograms per gram creatinine) (Basu et al. 2005). Of the 3,364 genotyped HEALS participants who passed QC, 2,053 had existing data on arsenic metabolites, as described previously (Ahsan et al. 2007). High-performance liquid chromatography (HPLC) was used to separate arsenobetaine, arsenocholine, iAs^V^, iAs^III^, MMA, and DMA (Reuter et al. 2003), and their concentrations were measured using inductively coupled plasma-mass spectrometry with dynamic reaction cell. Because As^III^ can oxidize to As^V^ during sample transport, storage, and preparation, we express total iAs as As^III^ + As^V^. iAs%, MMA%, and DMA% were calculated as percentages of the sum of urinary arsenic, after subtracting arsenobetaine and arsenocholine (forms of nontoxic organic arsenic from dietary sources) from total arsenic. Drinking water arsenic concentrations were analyzed by graphite furnace atomic absorption or, when concentrations were < 5 μg/L, by inductively coupled plasma-mass spectrometry (Cheng et al. 2004; van Geen et al. 2003).

*Ascertainment of skin lesions*. At baseline and at each follow-up interview of HEALS, skin lesions were ascertained using a structured protocol by trained study physicians. Through the whole-body examination, the study physician recorded the presence or absence of melanosis, leukomelanosis, and keratosis as well as their location, size, and shape. For the purposes of this analysis, skin lesion cases were defined as participants diagnosed with any type of skin lesion. In BEST, skin lesions were evaluated using protocols similar to those used in HEALS. All BEST participants had existing arsenic-related skin lesions at baseline.

*Estimation of variance in arsenic metabolism efficiency explained by SNPs (i.e., heritability)*. Our analysis sample was composed of 2,053 HEALS participants with data on genome-wide SNPs and arsenic metabolites. Because HEALS participants are selected from a relatively small geographic region, a subset of our participants are genetically related to another participant, as described previously (Pierce et al. 2012). We used the DMA% variable to represent arsenic metabolism efficiency because it is strongly and inversely correlated with both iAs% and MMA% and because DMA% showed the strongest association with 10q24.32 variants in our prior GWAS (Pierce et al. 2012).

To estimate the PVE in DMA% by genetic factors (i.e., the “heritability”), we used a linear mixed model (LMM) approach originally proposed by Yang et al. (2010). This method is often referred to as genomic restricted maximum likelihood estimation (GREML). The general purpose of the GREML method is to estimate the proportion of variation in a phenotype that is due to all measured SNPs. This is fundamentally different from the traditional GWAS approach because our goal is to estimate variance explained by all SNPs, as opposed to testing individual SNPs for association with a phenotype. The GREML method is well established, has been described in detail, and exploits the fact that genotypic similarity (i.e., “relatedness,” measured using SNPs) will be correlated with phenotypic similarity for phenotypes that are influenced by genetic variation. The GREML method can utilize data on very distantly related individuals, individuals that are typically considered “unrelated” in traditional GWAS. A LMM is used to estimate the PVE by measured SNPs for a phenotype, as implemented in the Genome-wide Complex Trait Analysis (GCTA) software package (Yang et al. 2011). For a detailed description of the analytic method, see Supplemental Material, “LMM Analysis.”

To quantify genetic similarity between individuals, we constructed an *n*-by-*n* genetic relationship matrix (GRM), where *n* is the sample size (*n* = 2,053) and each element represents the degree to which a pair of individuals are related. Each element of the GRM is the genome-wide proportion of alleles shared IBS (identical by state) between two participants, as described by Yang et al. (2011), referred to here as “K_IBS_.” Under circumstances where the individuals are closely related, K_IBS_ is a good estimate of allele sharing IBD, K_IBD_ (identical by descent, where the shared alleles are inherited from the same ancestor), because K_IBS_ will capture information on all variants in the genome. However, K_IBS_ is not an ideal estimate of K_IBD_ for distantly related individuals because it will primarily capture only information on measured SNPs (Zaitlen et al. 2013). Thus, SNP-based heritability estimates obtained from very distantly related individuals will tend to be lower than the true narrow-sense heritability.

Using the GREML method, we obtained three different types of PVE/heritability estimates. First we estimated PVE using all participants (using the full IBS-based GRM). Next, we estimated PVE using a modified GRM in which distant relatives were assumed to be unrelated (i.e., K_IBS_ values < 0.05 were set to zero), producing an estimate of the IBD-based GRM (Zaitlen et al. 2013). This provides an estimate of the full narrow-sense heritability (h^2^), which includes the additive effects of all genetic variation, including nongenotyped variants, but it is prone to bias due to shared environment. This h^2^ estimate is comparable to those generated in family-based heritability studies. We also estimated the PVE after excluding individuals from close-relative pairs to produce a data set of only distantly related individuals (all K_IBS_ < 0.05). This method provides an estimate of the heritability due to measured SNPs (h_g_^2^). The PVE estimate based on the full GRM (the first one described above) is essentially a mix of h^2^ and h_g_^2^. Covariates included in the LMM were age (continuous), sex (men vs. women), batch (batch 1 vs. 2, binary), water arsenic quartiles (categorical), smoking status (nonsmoker, former smoker, and current smoker, categorical), and body mass index (BMI; ≥ 10.2, 18.5–25.0, and ≥ 25.0 kg/m^2^, categorical). Twenty principal components (PCs; continuous) were included to minimize potential biases caused by population structure; PCs were generated using EIGENSTRAT (Patterson et al. 2006). PVE analyses were first run using only genotyped SNPs to construct the GRM, and then run again using both genotyped and imputed SNPs to construct the GRM.

*Regional heritability analysis*. We also conducted genome-wide regional heritability analysis using Regional Genomic Relationship Mapping (REACTA) software (Nagamine et al. 2012). This method quantifies the contribution of a specific genomic region to the heritability of a phenotype using a mixed model that includes random effects for a specific region and a residual whole-genome effect. The whole-genome additive effect was estimated by using all SNPs to construct the GRM, whereas the regional effect was estimated using only SNPs from a specific region to estimate a local GRM. We estimated the regional heritability across all 22 autosomes among all the non-close relatives (K_IBS_ < 0.05, *n* = 1,338). We analyzed 4,924 100-SNP windows for the genotyped SNPs (with an overlap of 50 SNPs between neighboring windows) and 4,787 300-SNP windows for the imputed SNPs (with an overlap of 50 SNPs between neighboring windows). *p*-Values for the heritability estimates were assessed using a Bonferroni-corrected *p* threshold (0.05/4,924 or 4,787 = 1.0 × 10^–5^).

*Polygenic scoring*. Because *AS3MT* variants that influence arsenic metabolism also influence arsenical skin lesion risk (Ahsan et al. 2006b; Pierce et al. 2013), we assessed the potential polygenic contribution of arsenic metabolism–related SNPs to skin lesion risk. We generated a polygenic model for DMA% using data from all 2,053 HEALS participants with arsenic metabolite data. Using this model, we generated SNP-based polygenic scores in an independent data set of 2,014 skin lesion cases (1,990 BEST samples and 24 HEALS samples) and 1,285 controls from HEALS, and we tested the score for association with case–control status. To ensure that our polygenic scoring analysis was not influenced by the contributions of highly correlated SNPs, we pruned out 170,512 SNPs to produce a data set of genotyped SNPs with no pairwise *r*^2^ values > 0.2 using the --indep-pairwise command in PLINK (http://pngu.mgh.harvard.edu/~purcell/plink/). To ensure we were evaluating associations for non-*AS3MT* SNPs only, we further excluded 36 SNPs within ± 1 Mb of the *AS3MT* transcribed region. We also removed 9,852 SNPs with low minor allele frequencies (MAF < 0.05), resulting in 77,347 SNPs that were included in the polygenic score analysis.

The polygenic analysis was conducted as follows. Among the 2,053 participants with DMA% data (the “training set”), we estimated a beta coefficient for the association between the minor allele of each SNP and DMA%, adjusting for age (continuous), sex, concentration of water arsenic (continuous), and genotyping batch (binary). For each individual in the case–control sample (the “testing set”), a polygenic score was calculated as follows: Using the results from the analysis of the training set, we first set a *p*-value threshold to select SNPs for inclusion in the polygenic model. Several *p*-value thresholds were used: 10^–4^, 10^–3^, 0.01, 0.1, 0.3, and 0.5. For each SNP with a *p*-value below the threshold, the number of minor alleles carried by each individual in the testing set (0, 1, or 2) was multiplied by the SNP’s beta coefficient derived from the training set. For each individual, these weighted allele counts were then summed over all SNPs passing the threshold and divided by the total number of summed SNPs to produce the polygenic score (as implemented in the PLINK “score” command) (Purcell et al. 2007). These scores were then tested for association with skin lesion status using mixed linear regression models adjusting for sex, age, and genotyping batch implemented in genome-wide efficient mixed model association (GEMMA) (Zhou and Stephens 2012). To approximate the corresponding odds ratio (OR), the beta coefficient was first divided by [*x*(1 – *x*)], where *x* is the proportion of cases in our sample, in order to estimate the beta from a logistic model. This quantity was exponentiated to obtain an OR.

*Analysis of candidate variants identified in prior studies*. We identified 20 variants in 15 genes with previously reported associations with arsenic metabolism phenotypes (Agusa et al. 2012; Breton et al. 2007; Chen et al. 2012; Chiou et al. 1997; Engström et al. 2010, 2011; Paiva et al. 2010; Porter et al. 2010; Rodrigues et al. 2012; Schläwicke Engström et al. 2009; Steinmaus et al. 2007). We examined their associations with arsenic metabolism phenotypes in our GWAS data using mixed linear regression models adjusted by sex, age, and genotyping batch. For those candidate SNPs that were not genotyped in our study, we identified proxy SNPs with *r*^2^ > 0.8 that were genotyped in our study based on HapMap2 CHB (Han Chinese in Beijing, China) and JPT (Japanese in Tokyo, Japan) data.

*Standard protocol approvals, registrations, and patient consent*. The study protocol was approved by the institutional review boards of The University of Chicago, Columbia University, and the Bangladesh Medical Research Council, and all study participants provided informed consent.

## Results

Characteristics of HEALS participants and their associations with DMA% are shown in Table 1. In a multivariate model, older age (> 50), female sex, and lower arsenic in either water or urine were associated with higher arsenic metabolism efficiency (higher DMA%). Compared with participants with BMI between 18.5 and 25.0, people of both higher and lower BMI had elevated DMA%. No association was observed for smoking status. BEST participants do not have DMA% data and were only involved in the polygenic scoring analyses; thus, these participants are not included in Table 1.

**Table 1:** Characteristics of HEALS participants and their associations with arsenic metabolism efficiency, that is, DMA% (*n *= 2,053).*^a^*

Characteristic	No. (%)^*b*^	DMA%		
		β	SE	*p*-Value
Sex
Women	1,015 (49.4)	Referent
Men	1,038 (50.6)	–2.98	0.41	< 0.0001
Age
17–29	438 (21.3)	Referent
30–39	589 (28.7)	–0.06	0.44	0.90
40–49	557 (27.1)	0.16	0.46	0.74
50–70	469 (22.8)	1.20	0.51	0.02
Water arsenic (μg/L)
Quartile 1 (0–8)	514 (25.3)	Referent
Quartile 2 (9–49)	503 (24.8)	–1.04	0.43	0.02
Quartile 3 (50–127)	507 (25.0)	–1.68	0.43	< 0.0001
Quartile 4 (128–864)	507 (25.0)	–2.57	0.43	< 0.0001
Smoking status
Never	1,161 (56.6)	Referent
Ever	892 (43.5)	–0.15	0.44	0.73
BMI (kg/m^2^)
10.2–18.4	864 (42.1)	Referent
18.5–24.9	1,059 (51.6)	0.89	0.32	0.005
25.0–51.8	130 (6.3)	2.22	0.65	0.0006
Urinary arsenic adjusted for creatinine (μg/g)
Quartile 1 (11–89)	426 (20.9)	Referent
Quartile 2 (90–176)	556 (27.2)	–0.19	0.44	0.66
Quartile 3 (177–343)	595 (29.2)	–1.25	0.43	0.004
Quartile 4 (344–8,556)	464 (22.7)	–2.74	0.46	< 0.0001
Prevalent skin lesion
No	1,974 (96.7)	Referent
Yes	67 (3.3)	–0.59	0.87	0.49
^***a***^β, SE, and *p*-values were obtained from mixed linear regression models, adjusting for age, sex, genotyping batch, smoking, BMI, and arsenic concentrations in drinking water. ^***b***^Categorical variables are presented as counts and percentages.				

Two types of PVE estimates for DMA% are presented in Table 2, those based on genotyped SNPs only, and those based on genotyped and imputed SNP. Below we discuss the results obtained using genotyped and imputed SNPs. The PVE estimate for DMA% was 16% (*p* = 0.08) when using a GRM calculated from all 2,053 participants. After adjusting for sex, age, concentration of water arsenic (quartiles), genotyping batch, BMI, and smoking status, the estimate decreased to 12% (*p* = 0.16). After adjustment for the top 20 principal components, the estimate changed to 15% (*p* = 0.10). The PVE estimate decreased to 5% after adjusting for two SNPs in the *AS3MT* region identified in our prior GWAS (rs9527 and rs11191527) (Pierce et al. 2012, 2013).

**Table 2:** Estimates of the percent variance explained (PVE) by genetic factors for DMA% obtained from linear mixed regression models.

HEALS participants	Covariate adjustment	All genotyped SNPs (*n *= 257,747)			All genotyped and imputed SNPs (*n *= 1,211,988)		
		PVE (%)	SE	*p*‑Value	PVE (%)	SE	*p*‑Value
All participants^*a*^ (*n *= 2,053)	No adjustment	13	10	0.09	16	12	0.08
	Adjusted for covariates^*b*^	10	10	0.15	12	12	0.16
	Further adjusted for PCs^*c*^	11	11	0.16	15	12	0.10
	Adjusting for two 10q24.32 SNPs	3	10	0.36	5	12	0.34
All participants, defining distant relationships as “unrelated”^*d*^ (*n *= 2,053)	No adjustment	48	13	0.0004	63	16	0.0002
	Adjusted for covariates^*b*^	42	14	0.002	54	17	0.001
	Adjusted for two 10q24.32 SNPs	35	14	0.007	41	17	0.01
PCs, principal components. ^***a***^Using the full GRM, K_IBS_ on all individuals. The PVE is in between the full narrow-sense heritability and the heritability due to measured SNPs. ^***b***^Covariates including sex, age (continuous), concentration of water arsenic (quartiles), genotyping batch, BMI, and smoking status. ^***c***^Twenty principal components as additional covariates to minimize inflation in significance testing caused by population stratification. ^***d***^Using a modified GRM, with K_IBS_ set as 0 if K_IBS_ < 0.05 (i.e., ignoring distant relationships); this approximates the K_IBD_ for all individuals. The PVE corresponds to the full narrow-sense heritability. After eliminating close relative pairs from the data set (K_IBS_ > 0.05), our sample size was too small (*n *= 1, 338) to generate a non-zero heritability estimate using GCTA.							

The PVE estimates for DMA% based on the modified GRM in which K_IBS_ < 0.05 were set to zero (i.e., based on all participants and defining distant relationships as unrelated) was 63% (*p* = 0.0002). After adjusting for covariates, the estimate decreased to 54% (*p* = 0.001). This estimate decreased to 41% (*p* = 0.01) after adjusting for the two SNPs in the *AS3MT* region. After eliminating close relative pairs from the data set (no K_IBS_ > 0.05), our sample size was too small (*n* = 1,338) to generate a non-zero heritability estimate using GCTA (data not shown).

**Figure 1:**
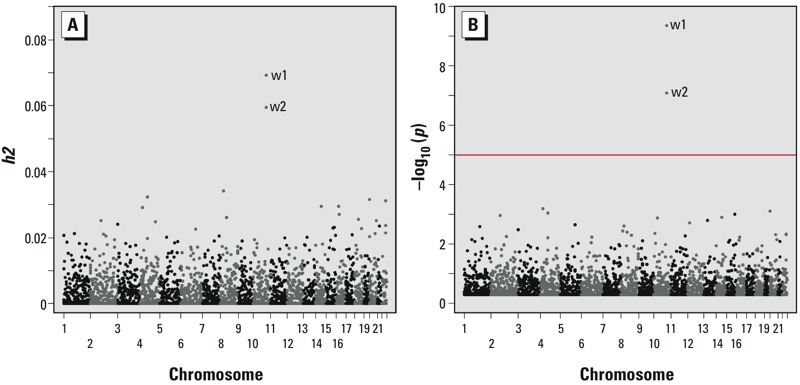
Regional heritability estimates (*A*) and corresponding *p*-values (*B*) for DMA%, excluding close relatives (K_IBS_ < 0.05, *n *= 1,338). Estimates were obtained using measured and imputed SNPs with a window size 100 SNPs with a 50 SNP overlap between windows. A total of 4,924 tests were conducted. The red line represents the Bonferroni-corrected *p*-value threshold. The two adjacent/overlapping windows that surpass the *p*-value threshold reside in the 10q24.32 region and are labeled “w1” and “w2.”

However, we were able to use the data set of distant relatives (no K_IBS_ > 0.05) to conduct regional heritability analysis. The most significant regional PVE estimates were obtained for two adjacent windows in the 10q24.32 region harboring *AS3MT*, and these accounted for approximately 7% of the variation in DMA% (*p* = 4.4 × 10^–10^ and 8.2 × 10^–8^) (Figure 1, w1 and w2). The regional heritability results based on genotyped data are the same as those based on imputed data (data not shown). After Bonferroni correction, no region showed a significant PVE estimate other than 10q24.32. Regional heritability analyses using the full data set (i.e., both close and distant relatives) produced very similar results (see Supplemental Material, Figure S1).

Polygenic scores for DMA% were not significantly associated with skin lesion status when using *p*-value thresholds of *p* < 10^–4^, *p* < 10^–3^, and *p* < 0.01 (unless including *AS3MT* SNPs when using a threshold of < 10^–4^); however, polygenic scores for DMA% were associated with skin lesion status when *p*-value thresholds of < 0.1, < 0.3, and < 0.5 were used to construct the score (Table 3). For example, when a threshold of *p* < 0.5 was applied, the beta coefficient for the association polygenic scores for DMA% was –0.05 (*p* = 0.02), suggesting that many alleles that cause very small increases in DMA% are also inversely associated with skin lesions. The beta coefficients (and ORs) in Table 3 correspond to a one standard deviation change in the polygenic score.

**Table 3:** Associations between polygenic scores for DMA% and skin lesion status.*^a^*

*p*-Value threshold	non-*AS3MT* SNPs					*AS3MT* SNPs included				
	No. of SNPs	Beta^*b*^	SE	*p*‑Value	OR (95% CI)^*c*^	No. of SNPs	Beta^*b*^	SE	*p*‑Value	OR (95% CI)^*c*^
*p *< 10^–4^	11	–0.007	0.007	0.34	0.97 (0.91, 1.03)	13	–0.02	0.007	0.01	0.93 (0.87, 0.98)
*p *< 10^–3^	87	0.001	0.008	0.89	1.00 (0.94, 1.07)	89	–0.005	0.008	0.53	0.98 (0.92, 1.05)
*p *< 0.01	801	0.01	0.01	0.22	1.06 (0.97, 1.15)	803	0.01	0.01	0.35	1.04 (0.96, 1.14)
*p *< 0.1	7,810	–0.03	0.02	0.04	0.87 (0.76, 0.99)	7,812	–0.04	0.02	0.03	0.86 (0.75, 0.99)
*p *< 0.3	23,281	–0.04	0.02	0.04	0.85 (0.73, 0.99)	23,283	–0.04	0.02	0.03	0.85 (0.73, 0.98)
*p *< 0.5	38,644	–0.05	0.02	0.02	0.82 (0.70, 0.96)	38,646	–0.05	0.02	0.01	0.82 (0.70, 0.96)
CI, confidence interval. ^***a***^The polygenic model was developed using all 2,053 participants with DMA% data and SNP data; the testing set was an independent set of 2,014 cases and 1,285 controls. ^***b***^The polygenic scores have been standardized, so the β coefficients from the mixed linear regression model correspond to a 1-SD change in the polygenic score, adjusted for sex, age, and genotyping batch. ^***c***^Odds ratios (ORs) were calculated by dividing the beta coefficient by [*x*(1 – *x*)], where *x* is the proportion of cases in our sample, in order to estimate the beta from a logistic model; this quantity was exponentiated to obtain an OR.										

Table 4 shows associations between arsenic metabolite percentages and variants that have shown suggestive evidence of association with arsenic metabolites in prior candidate gene studies. No SNP showed significant evidence of association (*p* < 0.05) except for *MTHFR*-rs1801133 (*p* = 0.03 for MMA%) and *DNMT1*-rs2228612 (*p* = 0.04 for DMA% and *p* = 0.03 for iAs%). The directionality of association was consistent with the prior publications for *MTHFR*-rs1801133, but *DNMT1*-rs2228612 showed an association in the opposite direction to the association previously reported.

**Table 4:** Association between arsenic metabolism phenotypes and candidate SNPs with associations reported in prior studies.

Gene	Reported SNP	Function	Population	Sample size	References	*p* for association^*a*^		
						DMA%	MMA%	iAs%
*GSTO1-1*	rs4925	Ala140Asp	Bangladesh	1,800	Rodrigues et al. 2012	0.46	0.94	0.60
			Taiwan	247	Chen et al. 2012
*GSTO2-2*	rs2297235	UTR-5	Bangladesh	1,800	Rodrigues et al. 2012	0.96	0.78	0.54
	rs156697	Asn142Asp	Chile	207	Paiva et al. 2010	0.51	0.72	0.55
*CHDH*	rs9001^*b*^	Glu40Ala	Argentina	111	Schläwicke Engström et al. 2009	0.51	0.23	0.79
	rs7626693	Intron	Argentina	111		0.28	0.19	0.44
*MTRR*	rs1801394^*c*^	Ile49Met	Argentina	111	
*GLRX*	rs3822751^*c*^	Intron	Argentina	111	
*PRDX2*	rs10427027	3’-UTR	Argentina	111		0.26	0.82	0.21
	rs12151144^*b*^	Intron	Argentina	111		0.26	0.82	0.21
*DNMT*	rs16999593	His97Arg	Argentina	111		0.15	0.59	0.11
*TXNRD2*	rs5746847^*b*^	Intron	Argentina	108	Engström et al. 2010	0.48	0.61	0.62
*Apex1*	rs1130409^*c*^	Asp148Glu	Argentina	108	
*GSTM1*	Gene deletion		Bangladesh	97	Breton et al. 2007
			Taiwan	115	Chiou et al. 1997
			Argentina	170	Steinmaus et al. 2007
*GSTT1*	Gene deletion		Taiwan	115	Chiou et al. 1997
*MTHFR*	rs1801133	C677T	Argentina	170	Steinmaus et al. 2007	0.053	0.03	0.20
	rs1801131	A1298C	Argentina	170		0.75	0.14	0.78
*GSTP1*	rs1695	Ile105Val	Vietnam	190	Agusa et al. 2012	0.85	0.52	0.49
*CBS*	rs234709^*c*^	Intron	Argentina	142	Porter et al. 2010			
	rs4920037	Intron	Argentina	142		0.25	0.21	0.50
*DNMT1*	rs2228612^*b*^	Intergenic	Bangladesh	361	Engström et al. 2011	0.04	0.31	0.03
*DNMT3B*	rs6087990	Intergenic	Bangladesh	361		0.66	0.15	0.61
*DNMT3B*	rs2424913	Intergenic	Bangladesh	361		0.46	0.19	0.97
^***a***^*p*-Values are based on a linear mixed regression model (GEMMA) to account for relatedness; adjustments include sex, age, and genotyping batch. ^***b***^Using rs2241807 data as a proxy of rs9001 (*r*^2^ = 0.81); rs10427027, rs5748485, and rs11672909 are proxies for rs12151144, rs5746847, and rs2228612 (*r*^2^ = 1.0); *r*^2^ values are based on HapMap GIH data. ^***c***^No data on tag SNPs was available for rs1801394, rs3822751, rs1130409, and rs234709.								

## Discussion

In this study, we have assessed, for the first time, the overall contribution of genetic variation to arsenic methylation capacity, as measured by DMA%, using SNP-based heritability methods. The PVE estimates obtained using only information on close relatives were 63%, consistent with estimates obtained from a recent family-based study (59%) (Tellez-Plaza et al. 2013). When distantly related individuals were included in the analysis, PVE estimates were much lower (16%). Overall, these results suggest that the excess heritability observed in studies of close relatives is due to variants not represented on the genotyping/imputing array (e.g., rare variants) or bias due to shared environmental factors. In regional heritability analyses, the *AS3MT* region produced the only significant PVE estimate. These results suggest that among common variants captured on our genotyping platform, *AS3MT* SNPs are the major genetic determinants of arsenic methylation capacity in this population and that contributions of other common variants to methylation capacity are substantially weaker than the effects of *AS3MT* variants.

Prior studies have examined familial aggregation patterns for arsenic methylation phenotypes. A study of Chileans with long-term exposure to high levels of arsenic in drinking water demonstrated that urinary concentrations of iAs, MMA, and DMA, as well as their ratios, were strongly correlated among siblings (*r* = ~ 80), after adjustment for total urinary arsenic (Chung et al. 2002). The authors observed lower correlations for father–mother pairs (*r* = 0.18), suggesting that genetic factors influence arsenic metabolic profiles. A population-based study in Taiwan found that patients with Blackfoot disease, an arsenic-induced peripheral vascular disease, were three times more likely to have a family history of Blackfoot disease than community controls (Chen et al. 1988), also suggesting that genetic factors influence arsenic metabolism and/or toxicity. Our heritability estimate for DMA% based on close relatives (48% or 63%) is similar to the heritability estimated in a recent study of Native American families (59%) (Tellez-Plaza et al. 2013).

The association between variants in the 10q24.32/*AS3MT* region with arsenic methylation capacity is consistent across many candidate gene studies (Agusa et al. 2011; Rodrigues et al. 2012; Schläwicke Engström et al. 2009) and has recently been confirmed in a GWAS (Pierce et al. 2012, 2013). In addition to *AS3MT*, dozens of candidate genes have been examined for association with arsenic methylation capacity in prior studies, based on various hypotheses related to methyltransferases, one-carbon metabolism, and reduction reactions (Schläwicke Engström et al. 2009). SNPs in *GSTO1, GSTO2* (Paiva et al. 2010; Rodrigues et al. 2012), *MTHFR* (Steinmaus et al. 2007), *PNP* (De Chaudhuri et al. 2008), *GSTM1* (Breton et al. 2007; Chiou et al. 1997; Steinmaus et al. 2007), and several other genes have even been reported to be associated with arsenic methylation capacity (Agusa et al. 2012; Engström et al. 2010, 2011; Ghosh et al. 2008; Hernández and Marcos 2008; Porter et al. 2010; Schläwicke Engström et al. 2009). However, many of these studies were limited by small sample sizes, and the genetic variants under investigation have not shown a great deal of consistency across studies (e.g., Ahsan et al. 2007; Hernández and Marcos 2008; Xu et al. 2009). In this study, we observed evidence of replication for only one SNP with a previously reported association (*MTHFR* rs1801133), and this association is very weak compared with SNPs in the 10q24.32 region. However, lack of replication could potentially be due to the fact that genetic variants can have different patterns of association in different populations because of population differences in linkage disequilibrium (LD) with causal variants, differences in allele frequency, and/or differences in the prevalence of environmental exposures that interact with the variant to influence the phenotype of interest.

In the present study, we used four different modeling approaches to estimate heritability (i.e., PVE). First, we estimated overall heritability using the full IBS-based covariance matrix for all study participants, including closely related individuals. This estimate should fall between the full narrow-sense heritability and the heritability due to measured SNPs (h_g_^2^). Second, we estimated heritability by focusing on close relatives, using only an IBD-based kinship matrix assuming zero relatedness between pairs of individuals whose estimated relatedness was < 0.05. This is an estimate of the full narrow-sense heritability (h^2^), capturing contributions of rare variants, but this estimate is prone to bias due to shared environmental factors. Third, we estimated heritability due to genotyped SNPs (h_g_^2^) using the IBS-based matrix constructed after removing close relatives from the data set. This is a more conservative approach to estimating heritability, as the presense of close relatives may cause bias due to shared environmental exposures. Fourth, we conducted regional heritability analyses, obtaining many heritability estimates corresponding to many small regions of the genome. Although the low heritability observed may reflect a limited contribution of common variants to arsenic methylation capacity, we do not have ideal power to accurately estimate modest heritability values. Excluding close relatives is an impotant consideration when conducting SNP-based heritiability estimation because relatives may be more likely to share similar (unmeasured) environmental exposures that influence the phenotype, potentially inflating heritability estimates (Yang et al. 2010). We have a substantial number of related individuals in our analysis, with only 1,338 samples remaining after removing related pairs with a relationship coefficient > 0.05.

The polygenic scoring analyses suggests that there may be common SNPs with weak effects on arsenic metabolism outside of the *AS3MT* region. For these analyses we assumed that SNPs influencing arsenic metabolism will also influence skin lesion risk. This assumption holds for DMA%-associated variants in the AS3MT region and is supported by multiple studies reporting an inverse association between DMA% and skin lesion risk (Ahsan et al. 2007; Gao et al. 2011; Kile et al. 2011; Lindberg et al. 2007; Pierce et al. 2013; Valenzuela et al. 2005). The observation that associations are present only when less stringent *p*-value thresholds are used implies that there are many variants with very weak effects on arsenic metabolism that also influence skin lesion risk. In order to identify such variants with very weak effects, association studies with larger sample sizes would be needed.

Arsenic-induced skin lesions are also influenced by many nongenetic factors, and we have assessed associations for several such factors in prior studies of this population. For example, we have reported that skin lesion risk is associated with arsenic, BMI (Argos et al. 2011), dietary patterns (Pierce et al. 2011), smoking, and occupational risk factors (Melkonian et al. 2011). Although these associations are clearly important as potential determinants of arsenic toxicity, we do not consider them in our polygenic scoring analysis because they are not potential confounders of the association between a SNP (or a SNP score) and skin lesion status.

In this work, we chose to use DMA% as a measure of arsenic methylation capacity. Alternative measures of methylation capacity include iAs%, MMA%, and metabolite ratios, which are highly correlated with DMA%. We chose to present results for DMA%, in part, because in our prior GWAS (Pierce et al. 2012), DMA% showed the strongest associations with SNPs in the *AS3MT* region compared with iAs%, MMA%, and metabolite ratios. Furthermore, PVE estimates for MMA% or iAs% were similar to those for DMA%, but somewhat weaker in magnitude (results upon request).

Although our study is the first SNP-based heritability study of arsenic methylation capacity, it has several limitations. First, our total sample size for metabolism study was only 2,053, which is relatively small for SNP-based heritability estimation. This hindered our ability to estimate heritability with high precision and to estimate heritability using a smaller, “unrelated” subset of study participants. Larger sample size, as well as denser SNP measurements (such as genome-wide sequencing), would enhance our ability to estimate heritability and conduct polygenic scoring analysis. We were able to measure arsenic metabolites only in urine and not in other relevant specimens such as blood, although this is a limitation of most studies of arsenic metabolism.

## Conclusions

In this SNP-based heritability study of arsenic metabolism efficiency, we estimated total narrow-sense heritability for DMA% to be 48–63% (using data on close relatives only), but the heritability due to measured SNPs was substantially lower (13–16%). Because the larger narrow-sense (“family-based”) estimate captures the effects of measured common variants and unmeasured rare variants (as well as shared environmental influences), and the smaller “unrelated” estimate captures the effects of measured common variants only, our results suggests that rare variants (e.g., AS3MT coding variants) and/or unknown or poorly measured environmental/lifestyle factors that cluster in families (e.g., dietary factors) make a substantial contribution of interindividual variation in arsenic methylation capacity. Moderate associations between a polygenic score for DMA% (composed of non-*AS3MT* SNPs) and skin lesion status were detected, suggesting the existence of additional common variants that have very weak effects on arsenic metabolism efficiency. Our regional heritability analyses did not detect additional susceptibility regions, consistent with the hypothesis that the effects of common variants outside of the 10q24.32/*AS3MT* region are likely to be very weak. Although these findings may not apply to other populations, our results suggest that future studies of Bangladeshi individuals with comparable exposure levels will have to have large sample sizes in order to detect associations between DMA% and common SNPs outside of the *AS3MT* region. Studies of rare variants may reveal genetic effects that contribute to the high heritability estimates observed in our family-based heritability analyses.

This work enhances our knowledge regarding the genetic architecture of arsenic methylation capacity in a population where the public health impact of arsenic exposure is substantial. Understanding the determinants of arsenic metabolism is critical because metabolism efficiency will likely affect the internal (or biological effective) dose, which will in turn impact risk for all arsenic-related health conditions. Understanding these determinants will improve our ability to identify high-risk subgroups and develop interventions to enhance metabolism efficiency or reduce exposure.

## Supplemental Material

(232 KB) PDF
