# Supplementary material for: The Genetic Architecture of Arsenic Metabolism Efficiency:A SNP-Based Heritability Study of Bangladeshi Adults
Source: Environ Health Perspect. 2015 Mar 13;123(10):985–92. doi: 10.1289/ehp.1408909 (PMC4590755; doi:10.1289/ehp.1408909)
Supplement: (232 KB) PDF [file ehp.1408909.s001.acco.pdf]

Environ Health Perspect
doi: 10.1289/ehp.1408909

Note to Readers: ***EHP*** **strives to ensure that all journal content is accessible to all readers. However, some figures and Supplemental Material published in *EHP* articles may not conform to 508 standards due to the complexity of the information being presented. If you need assistance accessing journal content, please contact ehp508@niehs.nih.gov. Our staff will work with you to assess and meet your accessibility needs within 3 working days.**

Supplemental Material

The Genetic Architecture of Arsenic Metabolism Efficiency: A SNP-Based Heritability Study of Bangladeshi Adults

Jianjun Gao, Lin Tong, Maria Argos, Molly Scannell Bryan, Alauddin Ahmed, Muhammad Rakibuz-Zaman, Muhammad G. Kibriya, Farzana Jasmine, Vesna Slavkovich, Joseph H. Graziano, Habibul Ahsan, and Brandon L. Pierce

Table of Contents

LMM Analysis

Reference

Figure S1. Regional heritability estimates (A) and corresponding P-values (B) for DMA% based on all samples (n= 2,053). Estimates were obtained using imputed SNP data with window size 300 SNPs with a 50 SNP overlap between windows. 4,787 tests were conducted. Red line indicates Bonferroni-corrected p threshold.

LMM Analysis

The LMM (linear mixed model) includes effects for all measured SNPs: $y=b_0+\sum w_i b_i +e$, where y is the phenotype of interest, b_i is the effect of the i-th SNP only, and w is a standardized genotype: $w_i= (z_i-2p_i)/ \sqrt{2p_i(1-p_i)}$, where z_i is an individual's minor allele count and p_i is the minor allele frequency, and e is the residual. Assuming Hardy-Weinberg equilibrium, the scaling allows SNP effects (b_i) to be treated as random variables from a distribution with variance σ_b^2. The analysis is implemented in an equivalent model, y= g + e, where g=Wb is a vector of genetic values calculated from an individual's genotype values and the variance of g is $WW'\sigma_b^2$. WW' is a matrix of pair-wise genetic relationships (genetic relationship matrix, GRM). The variance of the effects in vector g is equivalent to the variance explained by all SNPs in the original model that fits the SNP effects directly. This LMM allows us to estimate the overall heritability even though the number of SNPs exceeds the sample size, a situation in which typical linear regression would fail. The LMM was implemented in the Genome-wide Complex Trait Analysis (GCTA) software package (Yang et al. 2011).

Reference

Yang J, Lee SH, Goddard ME, Visscher PM. 2011. GCTA: A tool for genome-wide complex trait analysis. American journal of human genetics 88:76-82.

Figure S1. Regional heritability estimates (A) and corresponding P-values (B) for DMA% based on all samples (n= 2,053). Estimates were obtained using imputed SNP data with window size 300 SNPs with a 50 SNP overlap between windows. 4,787 tests were conducted. Red line indicates Bonferroni-corrected p threshold.

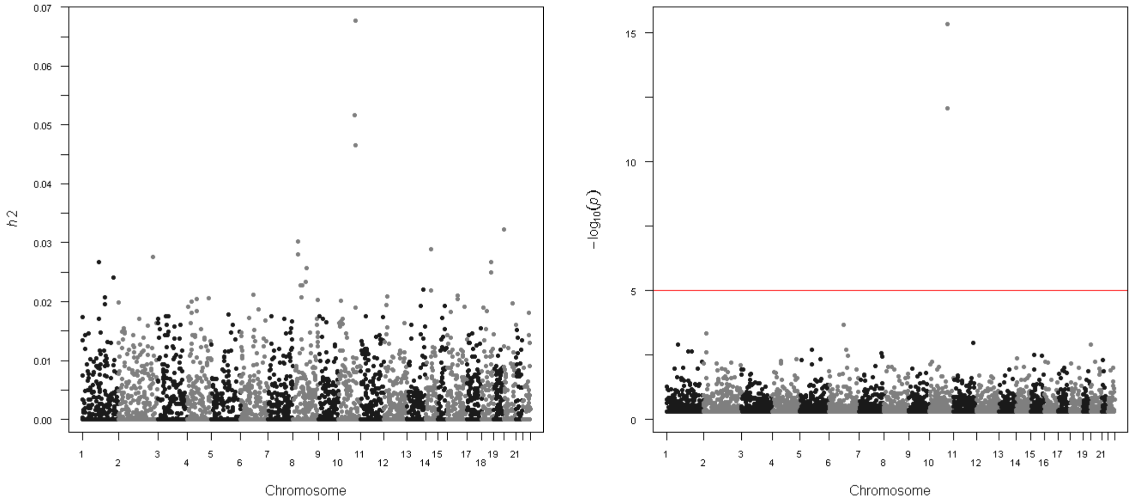

A B
